# Pressure-Induced Structural Phase Transition in Gd_2_Ce_2_O_7_ Oxide

**DOI:** 10.3390/ma19081615

**Published:** 2026-04-17

**Authors:** Tao Lv, Jia Qv, Limin Yan, Yan Li, Qiang Tao, Pinwen Zhu, Xin Wang

**Affiliations:** 1 State Key Laboratory of High Pressure and Superhard Materials, Jilin University, Changchun 130012, China; 2 College of Physics, Jilin University, Changchun 130012, China

**Keywords:** structural phase transition, the rare earth C-type structure, Gd_2_Ce_2_O_7_, high pressure

## Abstract

The high-pressure structural evolution of Gd_2_Ce_2_O_7_ has been studied by synchrotron X-ray diffraction (up to 31.8 GPa) and Raman spectroscopy (up to 38.9 GPa). A pressure-induced phase transition from cubic (space group *Ia-3*) to monoclinic (*C2*/*m*) crystal structure was observed to initiate at 11.3 GPa. This transition was primarily driven by the compression-induced cation rearrangement, which significantly altered the local coordination environment. The structural transformation proceeded via a gradual, continuous pathway and persisted up to the maximum experimental pressure of 31.8 GPa. Notably, the high-pressure phase remains stable upon complete decompression, confirming an irreversible structural transformation of Gd_2_Ce_2_O_7_.

## 1. Introduction

Ln_2_Ce_2_O_7_ (Ln = rare earth elements) has emerged as a pivotal material in energy science and catalysis due to its unique structural features and exceptional physical properties [1,2,3,4,5]. This compound belongs to the A_2_B_2_O_7_ solid solution family, where A represents a trivalent lanthanide and B is a tetravalent cation (Ce^4+^). Its structure is primarily governed by the ionic radius ratio of A^3+^ and B^4+^ cations [6]. When this ratio falls below 1.17, Ln_2_Ce_2_O_7_ adopts a disordered fluorite structure (accompanied by a rare earth C-type superstructure), as reported by Yamamura et al. [7]. This structural configuration is critical because it directly influences the material’s macroscopic properties, such as oxygen vacancy concentration and proton conductivity. Ln_2_Ce_2_O_7_ exhibits remarkable properties that make it suitable for advanced technological applications. Its high oxygen vacancy density enables efficient oxygen ion migration, resulting in superior proton conductivity [8]. This property is particularly advantageous for solid oxide fuel cells (SOFCs), where it can serve as a high-performance electrolyte, operating effectively at intermediate temperatures (300–600 °C) [9]. Additionally, its low thermal conductivity and high thermal expansion coefficient contribute to its stability under thermal stress, making it ideal for high-temperature catalytic processes [10]. The material’s catalytic activity is another key strength. Ln_2_Ce_2_O_7_ can act as an effective photocatalyst in the photodegradation of organic pollutants under light irradiation [11,12]. Meanwhile, it exhibits favorable hydrogen storage properties and has significantly contributed to reducing environmental pollution by using clean energy [13,14].

Gd_2_Ce_2_O_7_ is a promising candidate for electrochemical hydrogen storage devices due to its high surface area and chemical stability [15]. Moreover, its mechanical robustness and resistance to sintering at high temperatures (e.g., 800 °C) enable its use in durable coatings and structural components for extreme environments [16,17]. Despite these advantages, the structural evolution of Gd_2_Ce_2_O_7_ under external stimuli (such as pressure) remains poorly understood. Pressure plays a crucial role in structural engineering by enabling precise control over crystal structures, phase transition behaviors, and physical properties (such as electrical conductivity, magnetism, and catalytic activity) through altering the interactions between atoms or molecules within the material. In analogous A_2_B_2_O_7_ systems, pressure-induced phenomena are well-documented. Nd_2_Ir_2_O_7_ undergoes a pressure-induced magnetic phase transition near 10 GPa [18]. Eu_2_Ir_2_O_7_ exhibits a pressure-tuned insulator-to-metal transition [19]. Furthermore, Ho_2_Sn_2_O_7_ shows significantly enhanced photoluminescence due to pressure-driven structural modifications [20]. These precedents demonstrate the capacity of pressure to modify lattice parameters, shift defect equilibrium, and perturb the electronic band structure, thereby offering a direct pathway to tailor material properties. The specific mechanisms and critical pressure thresholds for Gd_2_Ce_2_O_7_ are still under investigation. This knowledge gap limits the optimization of its performance in practical applications.

This study systematically elucidates the high-pressure structural evolution of Gd_2_Ce_2_O_7_ through synchrotron X-ray diffraction and Raman spectroscopy. The structural and optical properties of Gd_2_Ce_2_O_7_ were investigated under hydrostatic compression up to 31.8 GPa and 38.9 GPa, revealing a phase transition near 11.3 GPa characterized by cation rearrangement accompanied by coordination environment modifications. Notably, the high-pressure phase remains stable upon complete decompression, confirming an irreversible structural transformation. These findings will not only enhance the fundamental understanding of high-pressure behaviors in Gd_2_Ce_2_O_7_ but also provide a theoretical basis for designing high-performance rare earth cerium oxide materials for energy and catalytic applications.

## 2. Materials and Methods

The high-purity Gd_2_Ce_2_O_7_ compound was synthesized via traditional solid-state reaction using stoichiometric mixtures of Gd_2_O_3_ (99.99% purity) and CeO_2_ (99.99% purity) with a molar ratio of 1:2 [21]. The homogeneous mixture was subsequently calcined at 1673 K for 12 h to ensure complete reaction. High-pressure measurements were conducted using a symmetric diamond anvil cell (DAC) system equipped with 400 μm diamond culet anvils. The sample containment was achieved by fabricating a 120 μm diameter cylindrical cavity within a pre-indented (thickness: 50 μm) T301 stainless steel gasket. Pressure calibration was systematically performed using the ruby fluorescence R1-line shift method, with silicone oil serving as the pressure-transmitting medium to maintain quasi-hydrostatic conditions [22,23]. All high-pressure experiments were carried out at ambient temperature.

The in situ high-pressure angle dispersive X-ray diffraction (ADXRD) experiments were systematically conducted at the BL04-MSPD beamline of ALBA synchrotron radiation facility (ALBA Synchrotron, Cerdanyola del Vallès, Spain), achieving a maximum pressure of 31.8 GPa. A monochromatic X-ray beam (λ = 0.4246 Å) was precisely focused to achieve a spot size of 20 × 20 μm (full width at half maximum, FWHM) on the sample surface. Diffraction data acquisition was performed using a Rayonix SX165 charge-coupled device (CCD) detector (Rayonix, LLC, Evanston, IL, USA). Prior to sample measurements, instrumental calibration was conducted using LaB_6_ standard powder to accurately determine the sample-to-detector distance and detector tilt angles. The acquired two-dimensional diffraction images were processed through DIOPTAS software (version 0.5.7) for integration into one-dimensional intensity versus 2θ profiles. For structural analysis, Rietveld refinement was conducted using the GSAS-II software package. We performed high-pressure Raman scattering experiments up to 38.9 GPa using a micro-Raman spectrometer (Renishaw, Wotton-under-Edge, UK) with 532 nm laser excitation. The backscattering geometry of the Raman signal was captured by a triple polychromator equipped with a volume transmission grating, with laser power stabilized at 50 mW to ensure excitation consistency. A laser beam was focused onto the sample surface via an Olympus objective lens (20.5 mm focal length, 0.35 numerical aperture), enabling precise irradiation. Raman spectral deconvolution was performed using a Gaussian fitting algorithm in OriginPro 2017, and characteristic peak positions were calibrated against standard reference spectra for spectral accuracy [24,25].

## 3. Results and Discussion

**Figure 1:**
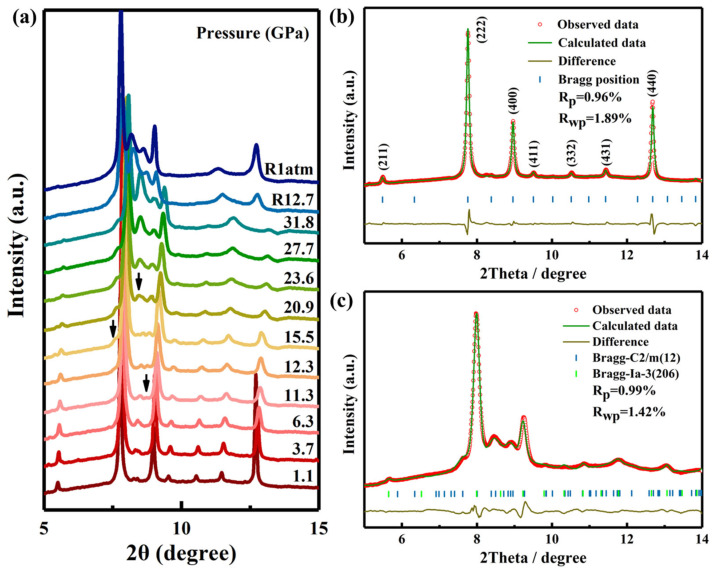
(**a**) Representative ADXRD patterns of Gd_2_Ce_2_O_7_ at various pressures. Black arrows denote high-pressure phase diffraction peaks. (**b**,**c**) present the Rietveld refinement results of Gd_2_Ce_2_O_7_ in the cubic phase (space group *Ia-3*) at 1.1 GPa and the monoclinic phase (space group *C2/m*) at 23.6 GPa, respectively. The brown line indicates the difference between the observed (red circles) and calculated (green solid line) profiles, while the blue and green vertical ticks mark the positions of the simulated diffraction peaks.

Synchrotron-based X-ray diffraction was employed to systematically investigate the phase stability of Gd_2_Ce_2_O_7_ under pressures up to 31.8 GPa, as illustrated in Figure 1a. For pressures below 11.3 GPa, the diffraction peaks were consistent with a C-type rare earth structure. With increasing pressure, all diffraction peaks exhibited a systematic shift toward higher 2θ values, wherein the initial peaks remained unchanged and no new peaks emerged. This behavior indicates a continuous decrease in the unit cell volume of Gd_2_Ce_2_O_7_ with increasing pressure, reflecting its intrinsic compressibility under high-pressure conditions. Starting at 11.3 GPa, a structural phase transition was observed in Gd_2_Ce_2_O_7_, marked by the emergence of additional diffraction peaks, the most intense of which appeared at ~8.4° and was attributed to the high-pressure phase. As pressure increased, the intensity of these newly formed peaks gradually intensified, indicating progressive development of the high-pressure phase. The pressure-induced transformation proceeded slowly and resembled phase transitions reported in other pyrochlore- or fluorite-structured oxides [21,26,27]. Notably, the original crystalline phase remained predominant throughout the compression cycle, indicating that the phase transition was still incomplete even at the maximum applied pressure of 31.8 GPa. Previous studies have shown that the C-type rare earth structure of Ho_2_Ce_2_O_7_ undergoes an irreversible phase transition under pressure [28]. Similarly, the high-pressure phase in Gd_2_Ce_2_O_7_ persisted upon full pressure release, confirming an irreversible structural transformation. These findings support a common phase transition mechanism among rare earth–cerium oxides under extreme conditions.

**Figure 2:**
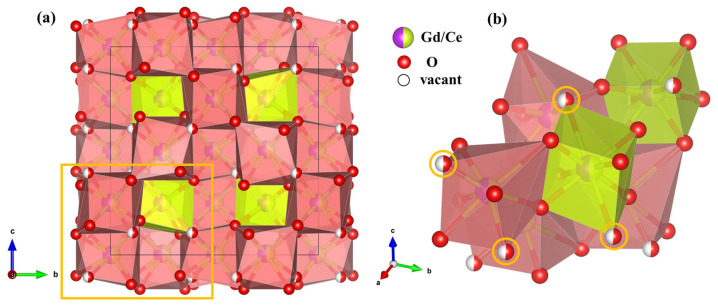
(**a**) The crystal structure of cubic Gd_2_Ce_2_O_7_ with rare earth C-type anion framework. (**b**) An enlarged view highlighting the local coordination environment within the marked region.

To gain deeper insight into the pressure-induced phase transition, the structural evolution of Gd_2_Ce_2_O_7_ under high pressure was investigated through Rietveld refinement of ADXRD patterns using the GSAS software suite. Figure 1b presents the refinement result at 1.1 GPa, showing excellent agreement between the observed and calculated profiles (Rp = 0.96%, Rwp = 1.89%). This confirms that the structure at this pressure is the C-type rare earth phase (space group *Ia-3*, No. 206) with a lattice parameter of a = 10.8502(9) Å, which aligns with the previously reported model for Gd_2_Ce_2_O_7_ [29]. The ambient-pressure crystal structure is illustrated in Figure 2a. In this cubic framework, rare-earth (Gd/Ce) cations co-occupy the 8b and 24d Wyckoff sites, coordinated by six or eight oxygen atoms. Oxygen atoms partially occupy the 16c and 48e positions. This arrangement results in statistically equivalent local oxygen environments for all cation sites, leading to a uniform spatial distribution and identical coordination geometry irrespective of the specific cation. Potential oxygen vacancy sites are located at the 16c Wyckoff position (x = y = z ≈ 0.452(5)), as shown in Figure 2b, where oxygen atoms exhibit partial and randomized occupancy. Under applied pressure, certain cubic pyrochlore or fluorite-related structures are known to transform into orthorhombic defect cotunnite-type phases [26,30]. A parallel can be drawn with Gd_2_O_3_, which undergoes a transition from a C-type rare earth structure to a monoclinic phase under compression [31]. For Gd_2_Ce_2_O_7_, the cubic symmetry (*Ia-3*) is retained from ambient conditions up to 11.3 GPa. At this threshold pressure of 11.3 GPa, the emergence of new diffraction peak marks the onset of a phase transition. These new peaks are indexed to a monoclinic phase with space group *C2*/*m*, having lattice parameters of a = 14.1188(8) Å, b = 3.6395(1) Å, and c = 8.3810(3) Å. The phenomena are analogous to the research of F. Zhang et al. [31]. Above 11.3 GPa, the diffraction patterns indicate the coexistence of the original cubic phase (*Ia-3*) and the newly formed monoclinic phase (*C2*/*m*). This two-phase mixture persists throughout the compression range studied, up to the maximum pressure achieved. The quality of the Rietveld fit for this coexistent region (e.g., Rp = 0.99%, Rwp = 1.42% at 23.6 GPa, see Figure 1c) supports the reliability of this structural model. All refined structural parameters are summarized in Table 1.

**Figure 3:**
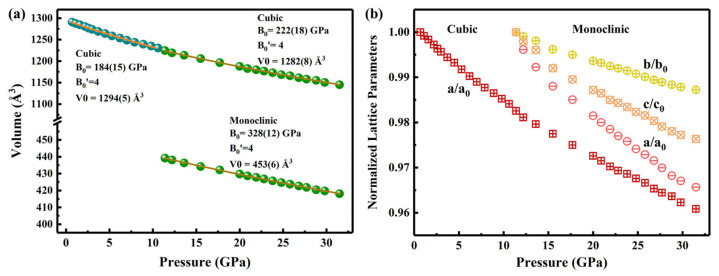
(**a**) Evolution of the unit cell volume of Gd_2_Ce_2_O_7_ under high pressure. The line corresponds to the fitted third-order Birch−Murnaghan equation of state. (**b**) Pressure-dependent relative normalized lattice parameters a/a_0_ (cubic) and a/a_0_, b/b_0_, c/c_0_ (monoclinic) for Gd_2_Ce_2_O_7_.

The lattice volume of Gd_2_Ce_2_O_7_ decreased monotonically with increasing pressure, exhibiting a distinct volume collapse at the phase transition pressure of ~11.3 GPa. The pressure-volume (P-V) data were analyzed using a third-order Birch–Murnaghan equation of state (EOS) [32,33], as shown in Figure 3a. For the cubic phase (P < 11.3 GPa), the EOS fitting (with the pressure derivative fixed at B_0_′ = 4) yielded a bulk modulus B_0_ = 184(15) GPa and an initial volume V_0_ = 1294 (5) Å^3^. This B_0_ value is slightly higher than that reported for the isostructural Ho_2_Ce_2_O_7_ (~170 GPa) [28]. For the high-pressure monoclinic phase (P ≥ 11.3 GPa), the analysis resulted in a substantially larger bulk modulus of B_0_ = 328(12) GPa (B_0_′ fixed at 4). The bulk modulus of Gd_2_Ce_2_O_7_ thus increases by approximately 78% upon transition to the monoclinic phase, signifying a dramatic reduction in compressibility. This marked stiffening trend is also observed in Ho_2_Ce_2_O_7_, underscoring a common mechanical response among these rare-earth cerium oxides under high pressure. The phase transition is accompanied by a massive volume shrinkage of ~64% (ΔV ≈ 779 Å^3^), calculated as Volume Collapse (%) = (1 − V_monoclinic_/V_cubic_) * 100% using unit cell volumes of the cubic (1224(4) Å^3^) and monoclinic (445(5) Å^3^) phases refined from high-pressure X-ray diffraction data at ~11.3 GPa. This extraordinary volume change far exceeds typical values reported in analogous oxide systems [26], and we attribute it to a unique reconstructive transformation involving significant bond cleavage and the complete reorganization of both anion and cation sublattices. Further insight into the compression behavior is provided by the lattice parameter evolution (Figure 3b). The cubic phase contracts isotropically, with its relative lattice parameter (a/a_0_) decreasing by 1.7% upon compression to 11.3 GPa. In stark contrast, the monoclinic phase exhibits strongly anisotropic compression in the 11.3–31.8 GPa range, with axial compression ratios of 3.4%, 1.3%, and 2.4% along the a-, b-, and c-axes, respectively.

**Figure 4:**
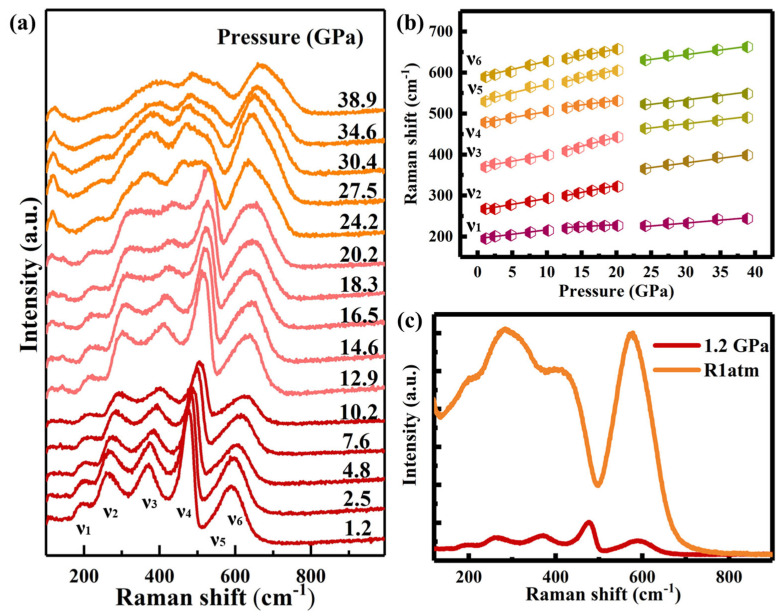
(**a**) records the Raman spectra of Gd_2_Ce_2_O_7_ under various pressures. (**b**) shows the pressure-dependent shift in Raman modes in Gd_2_Ce_2_O_7_. (**c**) displays the Raman spectra of Gd_2_Ce_2_O_7_ at 1.2 GPa and following full decompression to ambient conditions (R1atm) from 38.9 GPa.

The local structural response of Gd_2_Ce_2_O_7_ to high pressure was elucidated by in situ Raman spectroscopy at ambient temperature, with measurements extending to 38.9 GPa (Figure 4a). At a nominal pressure of 1.2 GPa, five vibrational modes were resolved at approximately 195 cm^−1^ (ν_1_), 267 cm^−1^ (ν_2_), 369 cm^−1^ (ν_3_), 478 cm^−1^ (ν_4_), 529 cm^−1^ (ν_5_) and 589 cm^−1^ (ν_6_). These spectral features are characteristic of the coexisting F-type (CeO_2_) and C-type (Gd_2_O_3_) structural domains, consistent with prior reports [28,34]. The observed breadth and intensity of these modes are ascribed to the inherent structural disorder and high oxygen vacancy concentration within the material [25,35,36]. Specifically, the ν_4_ mode was unequivocally assigned to the F_2_g symmetric stretching vibration of Ce-O bonds within eightfold-coordinated CeO_8_ polyhedra, a feature that serves as a definitive signature of the fluorite-derived CeO_2_ component, as reported by McBride et al. [37]. Identification of the C-type Gd_2_O_3_ structure was afforded by the ν_3_ mode, which originates from the (Ag + Fg) symmetric stretching vibration of Gd-O bonds in sixfold-coordinated GdO_6_ octahedra [36]. M. Coduri et al. reported that the ν_2_, ν_5_ and ν_6_ modes were associated with interactions between oxygen vacancies and their six next-nearest-neighbor oxygen atoms, whereas the ν_1_ mode arose from interactions involving oxygen vacancies and the four nearest-neighbor metal cations [34]. These spectroscopic assignments provide a crucial foundation for interpreting the pressure-induced evolution of the local structure and its correlation with the macroscopic phase behavior revealed by X-ray diffraction.

The monotonic blueshift of all Raman modes up to ~12.9 GPa (Figure 4b) corresponds directly to the continuous lattice contraction observed in the XRD-derived unit cell volume (Figure 3a) and the reduction in the a/a_0_ ratio for the cubic phase, both signatures of uniform bond shortening under hydrostatic stress prior to the structural instability. At 12.9 GPa, an anomalous increase in the intensity of Raman-active modes was detected, which may be correlated with the phase transition of Gd_2_Ce_2_O_7_ observed in the ADXRD measurements. The distinct alterations in the Raman spectra above 12.9 GPa—namely, the discontinuities in mode progression and the intensity redistribution favoring the ν_3_ (Gd-O) and ν_5_ (vacancy-related) modes—deliver compelling local-scale evidence for the phase transition. This spectroscopic signature aligns precisely with the emergence of new Bragg peaks in XRD patterns at ~11.3 GPa, which were indexed to a monoclinic (*C2*/*m*) high-pressure phase. Above 24.2 GPa, the ν_3_ mode (Gd-O) gains dominance over the ν_4_ mode, and the ν_5_ mode (oxygen vacancy-related) exhibits enhanced prominence. This evolution suggests that applied pressure promotes cation rearrangement and modifies local coordination geometries, thereby activating vibrational pathways associated with oxygen vacancy complexes. Furthermore, the persistence of the high-pressure Raman signature upon decompression (Figure 4c), particularly the maintained dominance of the ν_2_, ν_3_ and ν_5_ mode, offers independent, local-probe confirmation of the irreversible structural transformation previously established by the retention of the high-pressure XRD phase. Thus, the Raman spectroscopy results are fully congruent with and extend the diffraction-based narrative, offering a unified view of how pressure manipulates both long-range order and short-range interactions in Gd_2_Ce_2_O_7_, leading to an irreversible reconstructive transition.

## 4. Conclusions

This work investigated the crystal structure of Gd_2_Ce_2_O_7_ under high pressure, employing synchrotron X-ray diffraction (up to 31.8 GPa) and Raman spectroscopy (up to 38.9 GPa). Rietveld refinement of ADXRD revealed that the ambient-pressure cubic phase (space group *Ia-3*) remained stable up to 11.3 GPa. Beyond this pressure, a sluggish structural phase transition commenced, resulting in a two-phase mixture comprising the parent cubic phase (*Ia-3*) and a metastable monoclinic phase (space group *C2*/*m*). This mixed-phase state persisted throughout the compression range, indicating the transition remained incomplete even at the maximum pressure of 31.8 GPa. Complementary Raman spectroscopic analysis showed that, above 12.9 GPa, changes in vibrational modes signify a transition from ordered cations to a disordered state, accompanied by modifications in the local coordination environment. Crucially, the high-pressure phase was retained after complete pressure release, providing definitive evidence for an irreversible structural phase transition in Gd_2_Ce_2_O_7_.

## Figures and Tables

**Table 1:** The refined structural parameters of cubic phase and the high-pressure phase of Gd_2_Ce_2_O_7_ at 1.1 and 23.6 GPa, respectively.

Compounds	Gd_2_Ce_2_O_7_ (1.1 GPa)	Gd_2_Ce_2_O_7_ (23.6 GPa)	
Crystal System	Cubic	Cubic	Monoclinic
Space group	*Ia-3* (No. 206)	*Ia-3* (No. 206)	*C2*/*m* (No. 12)
a/Å	10.8502(9)	10.5553(6)	14.1188(8)
b/Å	10.8502(9)	10.5553(6)	3.6395(2)
c/Å	10.8502(9)	10.5553(6)	8.3810(3)
β/°			99.26
R_p_	0.96%	0.99%	
R_wp_	1.89%	1.42%	
R_exp_	2.28%	1.87%	
χ	0.83	0.76	
Atoms	Wyckoff (x y z)	Wyckoff (x y z)	Wyckoff (x y z)
Gd1/Ce1	24d (−0.0201(2) 0 0.25)	24d (−0.0138(2) 0 0.25)	4i (0.128(5) 0.5 0.483(9))
Gd2/Ce2	8b (0.25 0.25 0.25)	8b (0.25 0.25 0.25)	4i (0.179(2) 0.5 0.153(3))
Gd3/Ce3			4i (0.465(8) 0.5 0.213(2))
O (1)	48e (0.382(9) 0.153 (9) 0.348(2))	48e (0.354(7) 0.201(6) 0.392(5))	4i (0.125(9) 0 0.289(4))
O (2)	16c (0.452(5) 0.452(5) 0.452(5))	16c (0.528(7) 0.528(7) 0.528(7))	4i (0.318(2) 0.5 0.027(5))
O (3)			4i (0.295(4) 0.5 0.371(8))
O (4)			4i (0.468(2) 0 0.347(8))
O (5)			2b (0 0.5 0)

Note: The numbers in parentheses are the estimated standard deviations in units of the last digit.

## Data Availability

The original contributions presented in this study are included in the article. Further inquiries can be directed to the corresponding authors.
